# The Usability of Electronic Medical Record Systems Implemented in Sub-Saharan Africa: A Literature Review of the Evidence

**DOI:** 10.2196/humanfactors.9317

**Published:** 2019-02-25

**Authors:** Michael Kavuma

**Affiliations:** 1 Department of Tele-Health College of Health Sciences University of KwaZulu-Natal Durban South Africa; 2 MedLite Systems Limited Kampala Uganda

**Keywords:** review, computer systems, delivery of health care, sub-Saharan Africa

## Abstract

**Background:**

Electronic medical record (EMR) systems hold the exciting promise of accurate, real-time access to patient health care data and great potential to improve the quality of patient care through decision support to clinicians. This review evaluated the usability of EMR systems implemented in sub-Saharan Africa based on a usability evaluation criterion developed by the Healthcare Information and Management Systems Society (HIMSS).

**Objective:**

This review aimed to evaluate EMR system implementations in sub-Saharan Africa against a well-defined evaluation methodology and assess their usability based on a defined set of metrics. In addition, the review aimed to identify the extent to which usability has been an enabling or hindering factor in the implementation of EMR systems in sub-Saharan Africa.

**Methods:**

Five key metrics for evaluating EMR system usability were developed based on the methodology proposed by HIMSS. These were efficiency, effectiveness, ease of learning, cognitive load, and user satisfaction. A 5-point rating system was developed for the review. EMR systems in 19 reviewed publications were scored based on this rating system. It awarded 5 points per metric to any EMR system that was identified as excellent, 4 points for good, 3 points for fair, 2 points for poor, and 1 point for bad. In addition, each of the 5 key metrics carried a maximum weighted score of 20. The percentage scores for each metric were then computed from the weighted scores from which the final overall usability score was derived.

**Results:**

In possibly contributing to the usability of implemented EMR systems, ease of learning obtained the highest percentage score of 71% (SD 1.09) followed by cognitive load in second place with a score of 68% (SD 1.62). Effectiveness followed closely in third place at 67% (SD 1.47) and efficiency was in fourth place at 64% (SD 1.04). User satisfaction came in last at 63% (SD 1.70). The overall usability score for all systems was calculated to be 66%.

**Conclusions:**

The usability of EMR systems implemented in sub-Saharan Africa has been good with ease of learning possibly being the biggest positive contributor to this rating. Cognitive load and effectiveness have also possibly positively influenced the usability of EMR systems, whereas efficiency and user satisfaction have perhaps contributed least to positively influencing EMR system usability.

## Introduction

### Background

The free dictionary defines an electronic medical record (EMR) as a repository for active notations about a patient’s health; it is a computerized database that typically includes demographic, medical, laboratory, radiographic, drug, and other information about a patient [1]. EMR systems have evolved from pure record keeping to integrated enterprise-wide systems that hold the promise of accurate, real-time access to patient health care data while providing information necessary to improve patient care and lower costs [2]. Many institutions are developing integrated clinical workstations, which provide a single point of entry for access to patient-related, administrative, and research information. At the heart of the evolving clinical workstation lies the medical record in a new incarnation: electronic, accessible, confidential, secure, acceptable to clinicians and patients, and integrated with other nonpatient specific information [3].

EMR systems have also been shown to improve the quality of disease management, prevent disease-related comorbidities in hospitals [4], and to substantially reduce the risk of medication errors and adverse drug events [5]. They can significantly improve clinical documentation and medication refill turnaround time [6] and are perceived by physicians to have a positive impact on the quality of patient care [7]. The entire health care system can benefit immensely from the use of EMR systems with tangible benefits in cost savings and patient safety [8], making them especially relevant for low resource settings.

In Africa, electronic health care information systems have been driven mainly by the need to report aggregate statistics for government or funding agencies [9]. The use of computerized patient management systems is grossly limited in Africa where paper-based systems are still predominantly used in health care delivery. Some initiatives have been taken to deploy EMR systems though their focus has been heavily on HIV/AIDS care [10] and other infectious disease programs.

The Healthcare Information and Management Systems Society (HIMSS) defines a set of principles and methods for testing and evaluating EMR usability. It defines usability as the effectiveness, efficiency, and satisfaction with which specific users can achieve a specific set of tasks in a particular environment [11]. They submit that usability is possibly one of the most important factors hindering widespread adoption of EMRs and often has a strong direct relationship with clinical productivity, error rate, user fatigue, and user satisfaction. This literature review aimed at evaluating the usability of EMR systems implemented in sub-Saharan Africa using the usability evaluation criterion developed by HIMSS to identify the extent to which usability has enabled or hindered adoption of EMR systems in sub-Saharan Africa.

### Objectives

The objectives of the literature review were the following:

1. To evaluate EMR system implementations in sub-Saharan Africa against a well-defined evaluation methodology and assess their usability based on a defined set of metrics
2. To identify the extent to which usability has been an enabling or hindering factor in the implementation of EMR systems in sub-Saharan Africa

## Methods

### Evaluation Metrics

This literature review assessed EMR systems implemented in a sub-Saharan African context, using the evaluation methods and metrics proposed by HIMSS. HIMSS defines principles and proposes methods for evaluating and rating EMR usability. Its principles for good usability of EMR systems include simplicity, which refers to lack of visual clutter, concise information display, inclusion of only functionality that is needed to effectively accomplish tasks; they include naturalness, which refers to how automatically familiar and easy-to-use the application feels to the user; they also include consistency, which refers to how much an applications structure, interactions, and behaviors match a user’s experience with other software applications and how an application uses concepts, behavior, appearance and layout consistently throughout.

The principles also include minimizing cognitive overload by presenting all the information needed for the task at hand and displaying information organized by meaningful relationships: efficient interactions within the system, which refers to minimizing the number of steps it takes to complete tasks and providing shortcuts to experienced users and frequently used functions, incorporating forgiveness and feedback within the EMR system design, effective use of language in a form that is concise and unambiguous, effective information presentation in the appropriate density, and preservation of context by keeping screen changes and visual interruptions to a minimum.

From these principles, HIMSS proposes 5 key metrics for evaluating EMR system usability. These include efficiency, effectiveness, ease of learning, cognitive load, and user satisfaction. Efficiency as a test metric is defined as the speed at which a user can successfully accomplish the task at hand within the EMR system, whereas effectiveness is defined as the accuracy and completeness with which a user can achieve task goals within the EMR system. Ease of learning is defined as the time it takes a user to reach a specified level of proficiency in the use of the EMR system, whereas cognitive load is defined by how intuitively information and functionality are presented within the application, minimizing thought interruptions to users as they perform tasks within the software application. User satisfaction is defined as a person’s subjective response to his or her interaction with the EMR system, and it can be evaluated through a Likert-scale rating system or system usability scale questionnaires.

### Review Rating System

A 5-point rating system was developed for this review to rate instances of EMR systems implemented in sub-Saharan Africa based on the 5 key metrics mentioned above. The rating for this review weighted all 5 metrics equivalently in determining usability of EMR systems. Table 1 shows the applied rating per metric in the testing of EMR system usability.

**Table 1:** Key usability metrics used for the literature review and their maximum assigned weighted scores along with the 5-point rating system.

Key usability metric	Maximum weighted score	5-point individual usability rating for all metrics
Effectiveness	20	Excellent=5 points; Good=4 points; Fair=3 points; Poor=2 points; Bad=1 point
Efficiency	20	Excellent=5 points; Good=4 points; Fair=3 points; Poor=2 points; Bad=1 point
Ease of learning	20	Excellent=5 points; Good=4 points; Fair=3 points; Poor=2 points; Bad=1 point
User satisfaction	20	Excellent=5 points; Good=4 points; Fair=3 points; Poor=2 points; Bad=1 point
Cognitive load	20	Excellent=5 points; Good=4 points; Fair=3 points; Poor=2 points; Bad=1 point
Total	100	—^a^

^a^Not applicable.

For each of the 5-key metrics, a 5-point usability rating was applied to each individual metric and EMR systems rated based on how the authors of a publication about an EMR system described the performance of the system in their publication. For instance, if an EMR system was described as being excellent for any of the key usability metrics, then that EMR system was rated with 5 points for that metric. If it was described as good by the authors, then it was rated with 4 points, fair with 3 points, poor with 2 points, and bad with 1 point.

To illustrate, if an EMR system’s effectiveness was described as being excellent by the authors, then a usability rating of 5 points was assigned to that system’s publication for effectiveness. If the same system’s ease of learning was defined as poor by the authors, then the same system was assigned a usability rating of 2 points for ease of learning. Therefore, the highest usability rating that was attainable for any key metric was 5, whereas the lowest was 1. The search keywords and phrases were scored against this 5-point rating system in a uniform manner across all 5 metrics. Table 2 shows the uniform rating applied to keywords in the review.

In addition, in the methodology developed for this review, each metric carried a maximum weighted score of 20 per reviewed system, that is, effectiveness 20, efficiency 20, ease of learning 20, user satisfaction 20, and cognitive load 20. The rating for each system in each key metric was then computed as a score of the weight of that metric, that is, if a system was rated as fair in effectiveness by the authors, it was assigned a usability rating of 3 for effectiveness equating to a weighted score of (3/5)x20=12 for effectiveness. The same rating and scoring system was assigned to EMR system publications across all 5 key usability metrics.

The total score for all reviewed systems in each metric was then computed by summing up the weighted scores of the systems scored for that metric. The number of systems scored per metric was noted and the maximum attainable total score per metric was then calculated by multiplying the maximum weighted score of that metric by the number of systems scored in that metric. The percentage score for each metric was then calculated by dividing the total weighted score of that metric by the maximum attainable total score of the same metric and multiplying the result by 100%.

**Table 2:** Uniform rating of keywords against the 5-point rating system.

Scoring of search keywords	Effectiveness	Efficiency	Ease of learning	User satisfaction	Cognitive load
Excellent=5 points	Enhanced patient care and management	Totally eliminated delays	Quick user proficiency	Preferred system, viewed system as essential	Inclusion of standard treatment guidelines
Good=4 points	Significant improvement and system indispensable	Reduced patient or provider burden	User friendly interfaces, easy to comprehend, similarity with paper forms	Happy with system, user enthusiasm, rely on system, many perceived benefits from system use	Easily discerned functionality, well organized information, logical and systematic documentation
Fair=3 points	Effective, met objectives, improved data quality or records availability, decision support	Efficient, reduced time, streamlined procedures, and improved workflow	Easy to learn, simple, easy to use, and language customization	User satisfaction, acceptability, some benefits from use, limited adoption challenges	Intuitive, easy access to system information, and availability of reports
Poor=2 points	Functionality limitations and low usage	Increased time or burden	Complicated interfaces	User dissatisfaction and adoption challenges	Cluttered information and disorganized information
Bad=1 point	Did not meet objectives, ineffective, led to errors	Complicated workflow	Extensive effort to gain user proficiency	Hated system, perceived no benefit from use of system	Complicated access to functionality

**Table 3:** Grading ranges for overall electronic medical record system usability.

Percentage score range	Overall grading
80-100	Excellent
60-79	Good
40-59	Fair
20-39	Poor
0-19	Bad

For example, if the total score for all systems reviewed for effectiveness was calculated to be x and the number of systems scored for effectiveness was y, the maximum attainable total score for effectiveness was calculated as (yx20). The percentage score for effectiveness was then calculated as (x/[yx20])x100%. The same was applied across all 5 metrics to get the percentage scores for each metric. A 95% CI was applied to the percentage score of each metric.

The overall usability percentage score for EMR systems implemented in sub-Saharan Africa was then calculated as the sum of the total weighted scores of all 5 metrics divided by the sum of the maximum attainable total scores of all 5 metrics and the result expressed as a percentage. Finally, a predefined grading system of 5 ranges was applied to the overall percentage score to determine the overall usability performance of the reviewed EMR systems. The final overall usability of implemented EMR systems was graded based on Table 3.

### Search Criteria

**Figure 1:**
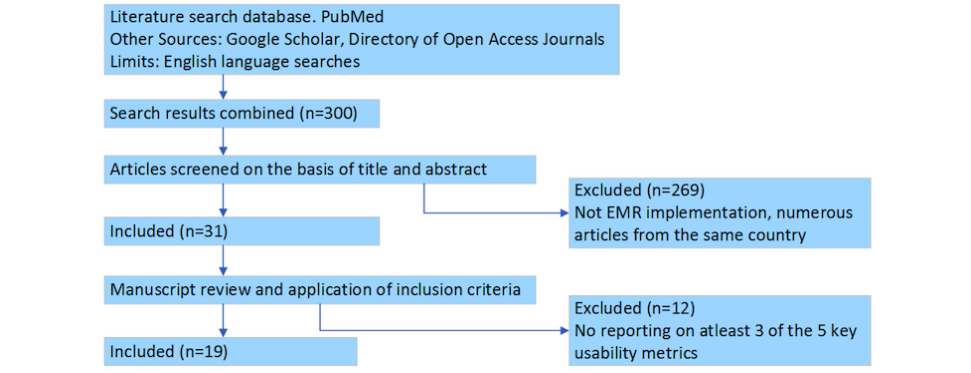
Preferred Reporting Items for Systematic Reviews and Meta-Analyses (PRISMA) flow diagram for identification, screening, and final inclusion of articles in the literature review. EMR: electronic medical record.

The literature for this review was obtained from searches in PubMed, Google Scholar, and the directory of open access journals in which 300 articles and literature published between the years 2000 and 2016 were reviewed. Of these, 19 articles were identified to meet the requirements for the literature review and were selected for review. The search terms used for the literature review included the following: implementation of EMR systems in sub-Saharan Africa, evaluation of EMR systems in Africa, computerized patient management systems in Africa, computerized hospital information system in Africa, health information systems in sub-Saharan Africa, testing or implementing electronic health record systems in Africa, and computerized clinic patient system in Africa. Country names from sub-Saharan Africa were also included in the search terms and appended to the ends of the search terms, replacing the words Africa or sub-Saharan Africa for some searches. Google translate was used to translate some French and Portuguese documents. Figure 1 shows a preferred reporting style flow diagram for systematic reviews and meta-analyses, showing the number of articles identified for the review, screened for eligibility, and finally included in the review.

A mixed-methods research approach was adopted for the literature review and involved both qualitative and quantitative research methods. The qualitative aspect focused on identification and extraction of keywords, phrases, and themes related to the 5 key usability metrics from articles included in the final review. The quantitative aspect focused on rating and scoring the systems in these articles using the 5-point rating and weighted scoring systems. Quantitative analysis was subsequently performed on the scores for each system in relation to the research objectives to identify the extent to which usability has been an enabling or hindering factor in the implementation of EMR systems in sub-Saharan Africa. Table 4 lists the 19 publications identified to meet the requirements of the literature review. Multimedia Appendix 1 shows the matching keywords identified in each publication for the 5 metrics. Tables 5 and 6 show the rating and scoring of each of the 19 systems in the 5-key metrics.

**Table 4:** List of publications identified to meet the requirements for the literature review.

Number	Publication	Software	Country	Focus
1	A global approach to the management of EMR (Electronic Medical Records) of patients with HIV/AIDS in Sub-Saharan Africa: the experience of DREAM Software [12]	DREAMS	Mozambique; Malawi: Tanzania; Kenya; Guinea; Republic; Guinea Bissau; Cameroon; Congo; Democratic Republic of Congo; Angola; Nigeria	HIV/AIDS
2	An electronic health record for infertility clinics [13]	EHRIC	South Africa	Reproductive health
3	An Electronic Patient Referral Application: A Case Study from Zambia [14]	ZEPRS	Zambia	Perinatal care
4	Combining Vital Events Registration, Verbal Autopsy and Electronic Medical Records in Rural Ghana for Improved Health Services Delivery [15]	MGV-Net VRVA	Ghana	Birth registration
5	Comprehensive Evaluation of Electronic Medical Record System Use and User Satisfaction at Five Low-Resource Setting Hospitals in Ethiopia [16]	SmartCare	Ethiopia	HIV/AIDS, Tuberculosis, and Pediatric Care
6	Designing and implementing an electronic health record system in primary care practice in sub-Saharan Africa: a case study from Cameroon [17]	MEDCAB	Cameroon	Primary health care
7	Electronic Patient Management System ePMS-Zimbabwe Collecting and Managing Data at the Patient Level for Better Treatment and Care [18]	ePMS	Zimbabwe	HIV/AIDS and Tuberculosis
8	Evaluation of Hospital Information System in the Northern Province in South Africa [19]	HIS	South Africa	General care
9	Experience Implementing Electronic Health Records in Three East African Countries [20]	OpenMRS	Kenya; Uganda; Tanzania	HIV/AIDS
10	Impact of an electronic clinical decision support system on workflow in antenatal care: the QUALMAT eCDSS in rural health care facilities in Ghana and Tanzania [21]	QUALMAT eCDSS	Ghana; Tanzania	Antenatal care
11	Implementation of a Cloud-Based Electronic Medical Record to Reduce Gaps in the HIV Treatment Continuum in Rural Kenya [22]	Uamuzi Bora	Kenya	HIV/AIDS
12	Implementation of Provider-Based Electronic Medical Records and Improvement of the Quality of Data in a Large HIV Program in Sub-Saharan Africa [23]	IDI ICEA	Uganda	HIV/AIDS
13	Implementing OpenMRS for patient monitoring in an HIV/AIDS care and treatment program in rural Mozambique [24]	OpenMRS	Mozambique	HIV/AIDS
14	Improvement of Service Capabilities Following the Establishment of an Electronic Database to Evaluate AIDS in Central Africa [25]	IeDEA DMS	Burundi; Cameroon; Democratic Republic of Congo	HIV/AIDS
15	Integration of ICT In Health Service Management in Heal Africa Hospital in DRCongo [26]	HEAL HMS	Democratic Republic of Congo	Primary health care and general care
16	OpenMRS Ebola Case Study [27]	OpenMRS	Sierra Leone	Ebola
17	Scale-up of networked HIV treatment in Nigeria: Creation of an integrated electronic medical records system [28]	FileMaker Pro EMRS	Nigeria	HIV/AIDS
18	Using Electronic Medical Records for HIV Care in Rural Rwanda [29]	OpenMRS	Rwanda	HIV/AIDS
19	Using Touchscreen Electronic Medical Record Systems to Support and Monitor National Scale-Up of Antiretroviral Therapy in Malawi [30]	POC EMR	Malawi	HIV/AIDS

**Table 5:** Rating of keywords for the 19 systems on the 5 key metrics.

Publication	Effectiveness		Efficiency		Ease of learning		User satisfaction		Cognitive load	
	Rating^a^	Maximum Weighted Score=20 points	Rating^a^	Maximum Weighted Score=20 points	Rating^a^	Maximum Weighted Score=20 points	Rating^a^	Maximum Weighted Score=20 points	Rating^a^	Maximum Weighted Score=20 points
Nucita, 2009 [12]	4	16	3	12	3	12	4	16	—^b^	—
Coetsee, 2014 [13]	3	12	3	12	—	—	3	12	—	—
Darcy et al, 2010 [14]	3	12	3	12	3	12	4	16	3	12
Ohemeng-Dapaaha et al, 2010 [15]	3	12	3	12	—	—	2	8	—	—
Tilahun and Fleur, 2015 [16]	2	8	2	8	3	12	2	8	4	16
Kmadjeu at al, 2005 [17]	3	12	3	12	3	12	3	12	4	16
United Nations Development Programme, 2014 [18]	3	12	4	16	—	—	4	12	3	12
Mbananga et al, 2002 [19]	3	12	3	12	—	—	2	8	—	—
Tierney et al, 2010 [20]	3	12	3	12	—	—	4	16	—	—
Mensah et al, 2015 [21]	3	12	3	12	—	—	—	—	3	12
Haskew et al, 2015 [22]	5	20	—	—	4	16	—	—	3	12
Castelnuovo et al, 2012 [23]	5	20	3	12	4	16	3	12	3	12
Manders et al, 2010 [24]	3	12	3	12	4	16	3	12	—	—
Newman et al, 2011 [25]	3	12	4	16	4	16	3	12	3	12
Guylain et al, 2015 [26]	3	12	—	—	4	16	—	—	2	8
Open MRS, 2015 [27]	3	12	4	16	3	12	3	12	5	20
Chaplin et al, 2015 [28]	5	20	3	12	4	16	4	16	4	16
Amoroso et al, 2010 [29]	4	16	4	16	4	16	2	8	3	12
Douglas et al, 2010 [30]	3	12	—	—	3	12	5	20	4	16

^a^Rating: Excellent=5, Good=4, Fair=3, Poor=2, Bad=1.

^b^Not applicable.

**Table 6:** Overall scoring of the 19 systems on the 5 key metrics.

Scores^a^	Effectiveness (19 systems scored)	Efficiency (16 systems scored)	Ease of learning (13 systems scored)	User satisfaction (16 systems scored)	Cognitive load (13 systems scored)
Total weighted score	256	204	184	200	176
Maximum attainable total score (max. weight x no. scored)	380	320	260	320	260
Percentage score (total/max x 100)	67%	64%	71%	63%	68%

^a^Overall usability percentage score=(sum of total weighted scores/sum of max attainable scores)x100%=66%.

## Results

### Effectiveness

All 19 publications reviewed were rated for effectiveness. Effectiveness was translated to systems being able to enhance patient care and management, provide significant improvement or be indispensable, be effective, meet implementation objectives, improve data quality, improve records availability, or being able to provide decision support to users. From the 19 systems, 3 systems (16%) obtained a rating of excellent for effectiveness, 2 systems (11%) obtained a rating of good for effectiveness, whereas 13 systems (68%) obtained a rating of fair for effectiveness. 1 system (5%) obtained a rating of poor for effectiveness and no system obtained a rating of bad for effectiveness. A majority of the systems reviewed were therefore found to be good in effectively achieving their implementation objectives. The percentage score for effectiveness of all 19 systems was found to be *67%* (SD 1.47; 95% CI).

### Efficiency

In the literature review, efficiency was associated with eliminating delays, reducing patient or provider burden, reducing time, and streamlining procedures or improving workflows. Efficiency was rated for 16 out of the 19 systems reviewed. No system obtained a rating of excellent for efficiency out of all 16 systems. From the 16 systems, 4 systems (25%) obtained a rating of good for efficiency. The majority of the systems, 11 out of 16 (69%), obtained a rating of fair for efficiency. Furthermore, 1 system (6%) obtained a rating of poor for efficiency and no system was found to be bad in efficiency. The percentage score for efficiency of the 16 systems scored was found to be *64%* (SD 1.04).

### Ease of Learning

Ease of learning was associated with quick user proficiency, user-friendly interfaces, easy system comprehension, similarity with paper forms, system ease of learning and use, as well as availability of language customization capabilities in the reviewed EMR systems. A total of 13 out of the 19 systems reviewed were rated for ease of learning. Out of the 13, no system obtained a rating of excellent for ease of learning. The majority of the systems, 7 out of 13 (54%), obtained a rating of good for ease of learning, whereas the rest of the systems, 6 out of 13 (46%), obtained a rating of fair for ease of learning. Ease of learning obtained the highest percentage score of all the 5 key usability metrics with a score of *71%* (SD 1.09).

### User Satisfaction

A total of 16 out of 19 systems reviewed were rated for user satisfaction. User satisfaction was associated with mention that users preferred the system or viewed the system as essential, were happy with or enthusiastic about the system, relied on the system, perceived many benefits from use of the system; there was also little mention of EMR system adoption challenges. Out of the 16, 1 system (6%) obtained a rating of excellent for user satisfaction. A substantial number of systems, 5 out of 16 (31%), obtained a rating of good for user satisfaction. The majority of the systems, 6 out of 16 (38%), obtained a rating of fair for user satisfaction. A total of 4 systems (25%) obtained a rating of poor for user satisfaction and no system obtained a rating of bad for user satisfaction. The percentage score for user satisfaction was the lowest of all the 5 metrics with a score of *63%* (SD 1.70).

### Cognitive Load

A total of 13 out of the 19 systems were rated for cognitive load. Cognitive load was associated with inclusion of standard treatment guidelines, easy discernment of system functionality, well-organized information within the system, logical and systematic documentation within the system, intuitive design of the EMR system, easy access to system information as well as availability of reports within the EMR system. Of the 13 systems, 1 system (8%) obtained a rating of excellent for cognitive load, whereas 4 systems (31%) were rated as having good cognitive load. The majority of the systems, 7 out of 13 (54%), obtained a rating of fair for cognitive load. Furthermore, 1 system (8%) was rated as having poor cognitive load and no system was found to have bad cognitive load. Cognitive load was found to have a percentage score of *68%* (SD 1.62) for the systems reviewed.

### Total Scores Per Metric

**Figure 2:**
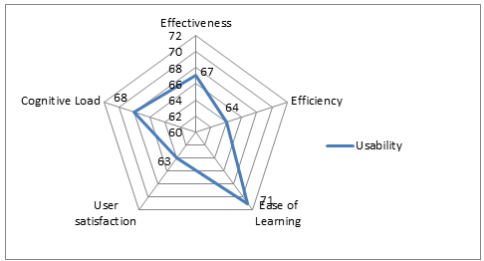
Radar graph showing usability of electronic medical record (EMR) systems implemented in sub-Saharan Africa. Ease of Learning has possibly positively influenced usability most.

The total percentage scores per metric were calculated as indicated in the results section above for each metric and plotted on a radar graph in Figure 2 to visualize their effect on EMR system usability.

### Overall Electronic Medical Record Usability Score

The overall usability score for EMR systems implemented in sub-Saharan Africa was calculated as the sum of the total weighted scores of all 5 metrics divided by the sum of the maximum attainable total scores of all 5 metrics and the result expressed as a percentage. It was found to be *66%.*

## Discussion

### Principal Findings

The usability of EMR systems implemented in sub-Saharan Africa has been good with an overall score of 66% across 5 key usability metrics. Ease of learning has possibly had the most positive influence on this rating and was defined for this review as the time it takes a user to reach a specified level of proficiency in the use of an EMR system. It probably has allowed users speedy and easy acquaintance with implemented systems, thereby enhancing their usability.

A relationship was observed between the scores for ease of learning and effectiveness in which a number of systems reviewed simultaneously obtained high scores in both metrics, suggesting that EMR systems might be more effective when they are easy to learn. A slight relationship was also observed between ease of learning and efficiency in which a few of the reviewed systems simultaneously obtained high scores in both metrics.

From the results, cognitive load has possibly contributed the second most to the usability of EMR systems with a percentage score of 68% (SD 1.62) for systems reviewed across the 5 key usability metrics. Cognitive load for this review was defined by how intuitively information and functionality are presented within the EMR system and appeared to have a slight relationship with user satisfaction.

Following closely in third place with a percentage score of 67% (SD 1.47) has been effectiveness in potentially positively contributing to EMR system usability in sub-Saharan Africa. Effectiveness was defined as the accuracy and completeness with which a user can achieve task goals within an EMR system, and it was found to have a relationship with ease of learning as described above and a slight relationship with user satisfaction.

Efficiency has probably contributed the second least to positively influencing the usability of EMR systems implemented in sub-Saharan Africa. Efficiency, defined as the speed at which a user can successfully accomplish the task at hand within the EMR system, obtained a percentage score of 64% (SD 1.04). A slight relationship was observed between efficiency and ease of learning as mentioned above, suggesting that efficiency benefits might be accrued from EMR systems that are easy to learn.

Finally, user satisfaction has probably contributed the least to positively influencing the usability of EMR systems implemented in sub-Saharan Africa. User satisfaction, which was defined as a person’s subjective response to his or her interaction with the EMR system, obtained a percentage score of 63% (SD 1.70). A slight relationship was observed between user satisfaction and effectiveness and user satisfaction and cognitive load as mentioned above, which might imply that where cognitive load is well-incorporated into the EMR system design, the systems are more likely to be effective and users are more likely to accept them.

### Conclusions

This literature review of the usability of EMR systems in sub-Saharan Africa used an evaluation methodology and usability metrics proposed by HIMSS to evaluate the implemented systems through a mixed-methods approach. The review identified that ease of learning has possibly had the most positive influence on the usability of EMR systems implemented in sub-Saharan Africa. Cognitive load and effectiveness have followed closely as second and third potential positive contributors to EMR system usability. Efficiency has possibly contributed the second least and user satisfaction probably contributed the least to EMR system usability.

Overall, usability appears to have been an enabling factor in the implementation of EMR systems in sub-Saharan Africa as it was found to be good in this review, and the approaches to incorporate usability into EMR implementations ought to prioritize ease of learning of the systems as this has been identified to potentially influence usability most. This supposition that ease of learning with 71% is the largest impact is true within the 95% CI because cognitive load at 68%+1.62=69.62 is below 71%−1.09=69.91 and therefore clearly distinct. Easy-to-learn EMR systems are possibly more effective as a relationship between ease of learning and effectiveness was identified in this literature review. Special attention also ought to be paid to user satisfaction while implementing EMR systems as this might not have been given adequate attention among the reviewed systems and therefore possibly contributed the least to the usability of EMR systems implemented in sub-Saharan Africa.

### Limitations of the Review

#### Methodology Limitations

This literature review of the usability of EMR systems implemented in sub-Saharan Africa was performed solely by the author and only evaluated EMR systems from publications and documentation about them. No physical evaluation or interaction with the actual systems was carried out as part of this literature review. Therefore, the review had limitations of not evaluating the EMR systems in their production clinical settings. The review also did not interview users of the systems to solicit their opinions on the usability of the systems. Moreover, only EMR system implementations where publications mentioned at least 3 of the 5 key usability metrics developed for this review were included in the final review.

#### Other Limitations

The review also equated EMR systems to patient management systems, clinical and hospital information systems, decision support systems, and electronic health record systems, and it did not take into consideration other confounding factors that might have influenced the usability of the reviewed systems such as hardware and related infrastructure, support and technical expertise availability, user engagement, funding, and so on. Therefore, the review only reviewed implemented EMR systems in their *already used and published about* state with a focus on their usability along the 5 key metrics with all other factors assumed constant.

## Appendix

Multimedia Appendix 1 Matching keywords identified in publications for the usability metrics.

## Abbreviations

EMR: electronic medical record
HIMSS: Healthcare Information and Management Systems Society
