# Supplementary material for: The Usability of Electronic Medical Record Systems Implemented in Sub-Saharan Africa: A Literature Review of the Evidence
Source: JMIR Hum Factors. 2019 Feb 25;6(1):e9317. doi: 10.2196/humanfactors.9317 (PMC6409508; doi:10.2196/humanfactors.9317)
Supplement: Multimedia Appendix 1 [file humanfactors_v6i1e9317_app1.pdf]

| Publication | Effectiveness; (matching keywords in publication) | Efficiency (matching keywords in publication) | Ease of learning (matching keywords in publication) | User satisfaction (matching keywords in publication) | Cognitive load (matching keywords in publication) |
|---|---|---|---|---|---|
| "Nucita A et al (2009) [12]" | Page 10: software has proved to be an effective tool; Page 12: an indispensable tool for the management of treatment | Page 12: procedures more efficient and the work of the center more streamlined | Page 9: Multilingual capabilities. System Screenshots show easy interface | Page 10: perceived benefits (improved data, test, patient and drug management) | —[a] |
| "Coetsee J et al (2014) [13]" | Page 2: improved data collection and record keeping. Page 2: Reduction in missing records | Page 2: definite saving in secretarial time | — | Page 2: bonus was that all the collected clinical data were accessible from anywhere | — |
| "Darcy N et Al (2010) [14]" | Page 15: system met the objectives established for it | Page 12: improved efficiency, and made information available locally | Page 11: staff found it easier to learn the referral application | Page 15: personnel's enthusiastic acceptance of this application | Page 10: provision of critical information to prepare for patients' arrival |
| "Ohemeng-Dapaaha S et Al (2010) [15]" | Page 4: integration of vital registration makes it possible for rapid assessment of effectiveness | Page 4: system is valuable for quickly identifying patterns. | — | Page 4: several challenges and limitations of the system were evident. | — |
| "Tilahun B & Fleur F (2015) [16]" | Page 6: usage of the system was found to be low. Only 2 of the core EMR functions were frequently used, unaware of 4 out of 12 core EMR functions | Page 5: strong disagreement towards the statements "The system helps me finish my task faster" | Page 2: The system also offers a touch screen interface to minimize the learning curve. | Page 6: The majority of them reported to be dissatisfied with the use of the system. | Screen shots: well organized information but with some visual clutter. |
| "Kamadjeu R et Al (2005) [17]" | Page 6: Although minor technical issues were identified, the system worked as intended | Page 6: a decrease in coding time, reduction of the duration of consultation and better management of patient flow was achieved | Page 5: simplicity, intuitiveness, and stability were the key concepts used in system development | Page 6: Users generally showed good acceptance of the system, measured by the comments expressed | Page 6: data from previous contacts readily available, easy generation of activity reports and statistics |
| "UNDP (2014) [18]" | Page 4: it improves the ability to track patients and follow up, increasing the likelihood of | Page 4: eliminated the multiple entry of patient information across different paper registers. | — | Page 5: so far most of our health care staff are very excited about the new system. | Page 4: easier analysis of the data, early warning indicators on the rate of patient survival. |

[a] — No data available

| | | | | | |
|---|---|---|---|---|---|
| | adherence to treatment | | | | |
| "Mbananga N et Al (2002) [19]" | Page 33: the clerks mentioned that the computer could easily trace the returning patients' records | Page 38: considered HIS more efficient in the registration and admission of patients | — | Page 28: reported that the system went offline several weeks and that caused backlogs in information updates | — |
| "Tierney W et Al (2010) [20]" | Page 5: could anticipate its personnel needs and avoid drug and lab stock outs | Page 5: Pulling charts for scheduled patients facilitated patient flow | — | Page 3: reception was generally positive.<br>Page 4: users were highly satisfied | — |
| "Mensah N et Al (2015) [21]" | Page 7: the positive influence on history taking and examinations is promising and may lead to better quality of care | Page 6: did not increase the duration of the ANC process at the study sites | — | — | Page 3: was developed to provide guidance and decision support at the point of care |
| "Haskew J et Al (2015) [22]" | Page 8: significant improvements in data quality and provision of clinical care, helping patients start HIV treatment early. | — | Page 3: electronic record replicates information in the national paper-based HIV outpatient record | — | Page 4: clinical decision support reminders were provided to the clinician |
| "Castelnuovo B et Al (2012) [23]" | Page 5: EMR led to a reduction in the total error rates from 66.5% to 2.1%. | Page 6: reduced the time spent with the providers filling forms. | Page 3: visually compelling and user-friendly | Page 5: EMR was rated Positively.<br><br>Page 6: our patients welcomed provider-based EMR. | Page 3: workflow management was built in. Screen shots: intuitive interface |
| "Manders E et Al (2010) [24]" | Page 4: EMR presented a clear advantage and the report generated from the electronic system proved more accurate. | Page 4: use the data to find patients who do not appear at the health facility in time to obtain their medication. | Page 6: system created to match the paper-based system, fully translated into Portuguese. | Page 4: clinicians with an active interest are using the system for ad-hoc analysis of patients in their care. | — |
| "Newman J et Al (2011) [25]" | Page 9: patient flow has been improved, data management system has facilitated scheduling and planning for follow-up visits. | Page 12: clinic visits more efficient<br><br>Page 9: Before, physicians were spending large amounts of time tallying these reports manually | Page 4: identical representation of the paper forms used by the clinicians | Page 12: Administrators and clinicians embraced the introduction of the electronic DMS. | Page 10: graphs of immunological progression for patients helpful for monitoring disease progression |

| | | | | | |
|---|---|---|---|---|---|
| "Guylain V et Al (2013) [26]" | Page 7: system was designed as specified and has improved service delivery | — | Page 3: user friendly menu from which the user can select the transaction; capture data and print reports | — | Screen shots provided show limited functionality |
| "OpenMRS.org (2015) [27]" | Page 3: worked with clinicians and staff to design a system that could work for their needs. | Page 3: UI is touch-based with large buttons for gloved hands, speed is of the essence since the suits are not breathable | Page 3: UI is touch-based with high contrast color schemes, large buttons for gloved hands, and large text | Page 3: Clinical users gave extensive feedback on their experiences with the system and how to improve it | Page 3: added Ebola-specific core concepts to simplify decision support and reporting. |
| "Chaplin B et Al (2015) [28]" | Page 10: system allowed program managers and staff to conduct quality control audits, which improved patientcare | Page 10: could more efficiently identify patients that required additional counseling. The EMRS also enabled efficient reporting | Page 7: A tabbed one page view. Referencing the most pertinent information on one screen is useful. | Page 7: Over time, the TRU has been very well received by program staff as well as patients. | Page 3: standard UI maintaining a consistent experience. Database was readily discernible. |
| "Amoroso CL et Al (2010) [29]" | Page 4: led to a 92% decrease in eight pre-defined data quality errors, from identification through EMR, HIV positive children are all receiving comprehensive care, with seven receiving ART | Page 4: A key benefit of the EMR system is the ability to automate real-time reports that would take many hours of work to compile under a paper-only system | Page 4: A user-friendly interface and automated weekly reminders have helped keep these tools in use | Page 5: Some clinicians used the system only infrequently | Page 5: Clinician access to information was addressed in increasing access to laboratory results and direct lookup of patient data |
| "Douglas G et Al (2010) [30]" | Page 3: The evaluation concluded that the system met both patient care and programmatic monitoring objectives | — | Page 2: touchscreen graphical user interface as a solution that was easy to learn and use | Page 3: 70% of the users expressed a preference for the touchscreen over the paper system; ongoing problems identified with the system that needed to be addressed | Page 3: guides the health care worker through a series of questions, one at a time and reports are ''active,'' allowing the user to tunnel down to a patient list from any indicator |
